# Murine Typhus Infection in Pregnancy: Case Series and Literature Review

**DOI:** 10.3390/pathogens10020219

**Published:** 2021-02-18

**Authors:** Melinda B. Tanabe, Lucas S. Blanton, Mauricio La Rosa, Camille M. Webb

**Affiliations:** 1 Department of Medicine, Division of Infectious Diseases, University of Texas Medical Branch, Galveston, TX 77555, USA; lsblanto@utmb.edu (L.S.B.); cmwebbca@utmb.edu (C.M.W.); 2 Department of Obstetrics and Gynecology, Division of Maternal Fetal Medicine, University of Texas Medical Branch, Galveston, TX 77555, USA; malarosa@utmb.edu

**Keywords:** murine typhus, endemic typhus, opossums, pregnancy, doxycycline, azithromycin, *Rickettsia typhi*, rickettsioses, fever in pregnancy, *Ctenocephalides felis*

## Abstract

Murine typhus is a flea-borne disease of worldwide distribution with a recent reemergence in the United States of America. There are limited data about the presentation, treatment, and outcomes in the pregnant population. We report on two cases of murine typhus during pregnancy and review the literature to compile previously reported cases. A comprehensive search was performed via the PubMed database for published articles between 1990 and 2020. Seven articles met the criteria of symptomatic pregnant murine typhus infection. A total of 37 patients were identified. Patients frequently presented with a prolonged duration of fevers prior to presentation, headache, and elevated hepatic transaminases. The diagnosis was predominantly based on serology. Treatment varied. Overall, the pregnancy outcome was favorable. Murine typhus can mimic other pregnancy-related pathologies. More exclusive and large-scale studies are needed to learn more of murine typhus during pregnancy.

## 1. Introduction

**Figure 1:**
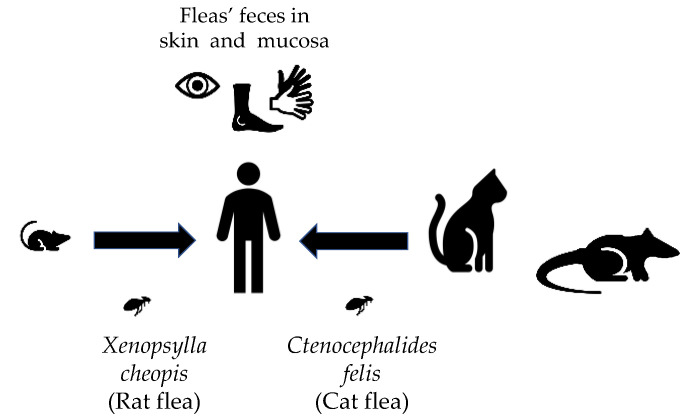
“*R. typhi* transmission cycle.” *R. typhi* can be transmitted via different vectors depending on the geographic location. The *R. typhi*-infected rat fleas are involved in the transmission via rats. Infected cat fleas have been found in opossums and cats. Both of the vectors play a role in the transmission to humans. Infection reaches the human via infected flea feces inoculated in mucosa or wounds.

Murine typhus (MT) is a flea-borne disease caused by *Rickettsia typhi* [1]. MT has a worldwide distribution, but most reported infections arise from Southeast Asia, Northern Africa, Australia, China, and North America [2]. It is known to be prevalent in cities and ports where rats (*Rattus* spp.) and their fleas (*Xenopsylla cheopis*) thrive [3]. In the United States, the disease is endemic in Texas, Southern California, and Hawaii. In the past few years, there has been a reemergence of *R. typhi* infections in the United States (USA), likely due to shifts in the reservoir and vector [4,5]. Whereas the rat flea is the primary vector throughout the world, the cat flea (*Ctenocephalides felis*) is the contemporary vector in the USA [4,6]. Prior studies on the prevalence of *R. typhi*-infected fleas in cats have been contradictory, but even the low infection rates cannot exclude the role of cats in the transmission of the disease [7]. In Texas, *R. typhi*-infected cat fleas are carried mainly by opossums [4,7] (Figure 1).

Infection is thought to be acquired when flea feces contaminated with the bacteria are inoculated into a wound (such as the flea bite) or mucous membranes (Figure 1). Other potential mechanisms are inhalation of infected flea feces or direct inoculation via a flea bite. The incubation period of most rickettsial diseases varies between 5 and 14 days. Infection in the general population tends to be mild, with patients presenting with an undifferentiated febrile illness. Severe manifestations, such as acute kidney injury, respiratory insufficiency, delirium, coma, and death may occur. Diagnosis is empirical, and it is confirmed by serology (usually with the indirect immunofluorescence assay (IFA)) demonstrating a fourfold increase in IgG (Immunoglobulin G) titers between acute- and convalescent-phase specimens. Studies from spotted fever group rickettsioses have shown that IgM is not more sensitive than the IgG isoform. IgM (Immunoglobulin M) is also subjected to higher cross-reactivity, making IgG more attractive for the diagnosis [8]. One of the main challenges regarding laboratory diagnosis is that reactive antibodies are usually not present during early illness [4]. Therefore, prompt clinical diagnosis and empiric treatment are important, as early treatment has been associated with good outcomes [9]. Other less common forms of diagnosis are polymerase chain reaction (PCR) amplification of rickettsial DNA and immunohistochemical demonstration of *R. typhi* from tissues or cultivation of the organisms in cell culture. Their high costs and need for specialized equipment limit their widespread use [8]. 

There have been efforts to alert physicians to the disease in the general population, as diagnosis relies on clinical suspicion. However, little is known about the incidence, presentation, treatment, and overall outcomes of MT during pregnancy. We herein present two cases of murine typhus during pregnancy from Texas. Subsequently, we reviewed the literature on all reported cases of symptomatic murine typhus during pregnancy in the past 30 years and summarized the clinical findings.

## 2. Case Series

### 2.1. Case Reports

#### 2.1.1. Case 1

A 36-year-old G7P4 30-weeks-pregnant patient, with a past medical history of prior pregnancies complicated by preeclampsia, and chronic headaches, presented with fevers and worsening headaches (HAs) of 1-day duration. The HAs were frontal, dull, without radiation, 8/10 in intensity, and partially improved with acetaminophen. She was from East Texas. The family history and medications were noncontributory. On admission, the temperature was 38.3 °C, blood pressure 104/76 mmHg, heart rate 116 beats per minute (bpm), respiratory rate 18 respirations per minute (rpm), and the oxygen saturation was 96% at room air. The physical exam was appropriate for gestational age. The laboratories were significant for leukocytosis (15,140 cells/mm^3^ with bands), relative thrombocytopenia (196,000 cells/mm^3^ from a baseline of 258,000 cell/mm^3^), and hyponatremia (serum Na 130 mml/L). Her blood cultures, influenza swab, and urine culture were negative. There were no abnormalities on the fetal parameters or fetal ultrasound. She was given supportive care. On hospital day (HD) 4, the patient developed worsening HA, fever (39.4 °C), and respiratory distress (saturation of 88–90% on 4 L of supplemental oxygen). Her blood work revealed worsening thrombocytopenia (138,000 cells/mm^3^) and an abnormal liver profile (alkaline phosphatase (ALK) 40 IU/L, alanine aminotransferase (ALT) 76 IU/L, and aspartate aminotransferase (AST) 100 IU/L). Computed tomography of the chest showed no pulmonary embolism but demonstrated diffuse septal thickening and patchy central ground-glass opacities with reactive lymphadenopathy. An echocardiogram was unremarkable. She was started empirically on furosemide for fluid overload and ceftriaxone plus azithromycin for community-acquired pneumonia on HD 6. A respiratory PCR panel was negative for common pathogens. On HD 9, the patient continued to be febrile. The Infectious Diseases Consultation Service recommended continuing with azithromycin and obtain serologies for murine typhus. Due to persistent headaches, a magnetic resonance imaging of the brain without contrast and a magnetic resonance venography were performed, showing no acute intracranial abnormality and no evidence of venous sinus thrombosis. A lumbar puncture was performed, and analysis of the cerebrospinal fluid (CSF) revealed pleocytosis (White Blood Cell count (WBC) 120 cells/μL, 44% neutrophils), hypoglycorrhachia (44 mg/dL), and an elevated protein level (81 mg/dL). The bacterial, fungal, and acid-fast bacilli cultures were negative. On HD 10, MT serology revealed an IgM titer of 1:1024 and an IgG titer of 1:512 (Rickettsia IFA IgM and IgG kit, Semi-Quantitative Indirect Fluorescent Antibody, Focus Diagnostics Inc., Cypress, CA, USA). The patient’s symptoms improved, and a course of azithromycin was completed. The subsequent serology, performed 27 days after the last serology, revealed an MT IgM titer of >1:1024 and an IgG titer of 1:256 compatible with probable but not definite, MT, as a fourfold change in titers could not be confirmed. She had no further complications during her pregnancy, delivering a healthy baby via cesarean section due to non-reassuring fetal heart tones at 39 weeks. 

#### 2.1.2. Case 2

A 20-year-old G1P0 patient at 12 weeks of gestation with poor prenatal care, presented with 7 days of fever, headaches, myalgias, and a maculopapular rash. She was from Southeast Texas, and she reported contact with a flea-infested stray kitten. She had no significant family or social history. She was not taking any medications. On admission, her temperature was 38.5 °C, heart rate 124 bpm, blood pressure 131/78 mmHg, respiratory rate 18 rpm, and the oxygen saturation was 97% at room air. The physical exam was relevant for mild bilateral cervical lymphadenopathy, right leg tenderness to palpation without edema, and a maculopapular rash on her upper extremities. The laboratories demonstrated hyponatremia (Na 128 mmol/L), hypokalemia (K 3.2 mmol/L), glucose 94 mg/dL, elevated transaminases (ALT 216, AST 152 IU/L), bandemia (WBC 7.86 cells/mm^3^ with bands), and thrombocytopenia (PLT 145,000 cells/mm^3^, baseline 216,000 cells/mm^3^). The serologies for parvovirus, toxoplasmosis, bartonellosis, CMV, HIV, syphilis, and viral hepatitis were non-reactive. The blood cultures were negative. The urinalysis revealed 17 WBC and mild proteinuria. An abdominal ultrasound showed a normal liver and gallbladder. A lower extremity ultrasound showed no deep venous thrombosis. The fetal ultrasound and hemodynamic parameters were within normal limits. A lumbar puncture was performed, and the laboratory studies of the CSF were unremarkable. On HD 4, the patient was empirically treated with doxycycline, and sera were tested for typhus group antibodies given high concerns for MT. Her fever, rash, and other symptoms resolved. The *R. typhi* IFA demonstrated an IgM titer of 1:512 and an IgG of 1:128 (Rickettsia IFA IgM and IgG kit, Semi-Quantitative Indirect Fluorescent Antibody, Focus Diagnostics Inc., Cypress, CA, USA). The convalescent serum obtained 15 days after, showed an IgM titer of 1:1024 and an IgG of 1:1024, confirming the diagnosis of murine typhus. She subsequently delivered a healthy baby at 39 weeks via cesarean section due to fetal intolerance to labor. 

## 3. Materials and Methods

A PubMed search was performed using the terms “murine typhus” “pregnancy”, “endemic typhus”, “*R. mooseri*”, and “*R. typhi*”. The only articles considered were those based on human symptomatic infection during pregnancy, electronically available in English, and published between January 1990 and November 2020. Exclusion criteria were asymptomatic murine typhus in pregnancy (based only on seropositivity) and undiagnosed febrile illness in pregnancy. Data were extracted based on the number of patients, demographics, past medical history, presentation, diagnosis, treatment, and overall pregnancy outcome after infection. Poor outcome was defined as stillborn, prematurity (<35 weeks), and fetal growth restriction (birth weight < 10th percentile). Full term was defined as delivery between 37–42 gestational weeks. Maternal comorbidity was defined as the presence of diabetes, hypertension, asthma, thyroid disorder, obesity, mental health conditions, co-infection with another pathogen (e.g., malaria, dengue, and scrub typhus), and substance and/or tobacco abuse [10]. Fever was defined as T > 38 °C. Lastly, hyponatremia was defined as < 134 mEq/L, absolute thrombocytopenia as PLT < 150,000 cells/mm^3^, and transaminitis as ALT > 40 IU/L. Information from the aforementioned cases was included in the analysis. A literature review was later performed based on available published data. 

## 4. Results

### Result Data

Seven articles were found describing MT during pregnancy. Of those, five were case reports [11,12,13,14,15,16,17], and two were based on observational population studies [15,16].

Data from the reported cases are presented in Table 1. A total of 37 pregnant patients with fever due to murine typhus were identified. Overall, there was heterogeneity in gestational age during infection, with 7/22 patients presenting during the first trimester. The origins of the populations of these reports were diverse: Southeast Asia (n: 29), USA (n: 6), Australia (n: 1), and the Middle East (n: 1). The age ranged from 17 to 36 years. Maternal comorbidities were present mainly in the Asian studies, corresponding to smoking and co-infections with malaria, dengue, pyelonephritis, and scrub typhus. Only a few studies reported risk factors for infection (3/7). 

All patients presented with fever (>38.5 °C), and the median for the duration of fever prior to admission/diagnosis was 7 days. They frequently presented with headache (21/22, 95%) and elevated liver enzymes (8/8, 100%). Diagnosis of MT was mainly based on serology, although some were positive by PCR. PCR results were exclusively reported from Asia, while cell-free next-generation sequencing was used twice in the USA. 

Treatment for MT varied. The use of erythromycin or ampicillin was noticed in earlier reports, while azithromycin and doxycycline were seen in later reports. From the Asian reports, eight cases were not treated and had spontaneous resolution with good outcomes. With the exception of the studies originating from Southeast Asia, there were no poor pregnancy outcomes noted in the USA, Australia, or Cyprus [11,12,13,14]. In the study by McGready et al., three patients had a poor outcome: one stillborn, one born prematurely, and two with low birth weight. Five patients in this study had other comorbidities (e.g., malaria co-infection, and smoking history) [15]. In the study by Chansamouth et al., a total of 15 patients were found with MT by serology or PCR. Most of the patients had a relatively uncomplicated pregnancy, except for two cases that had preterm births and coincidentally did not receive any anti-rickettsial treatments [16]. 

## 5. Discussion and Literature Review

Arthropod-related infections are on the rise globally. From 2004 until 2016, the overall reported cases in the USA have tripled, with a total of 642,602 cases [18]. The national prevalence of MT is not accurately known due to variable reporting and underdiagnosis. In Texas, there have been 3507 cases reported from 2008 to 2018, while in California, 1185 cases were diagnosed between 2001 and 2020 [5,19]. The numbers are expected to be even higher in Southeast Asia. Accurate epidemiological data are lacking, but prevalence studies in the area range from 0–21.5% [20]. Geographical, socio-economic, vector distribution, and increased interest in the field are some of the reasons for these findings [20]. However, due to a lack of accurate point-of-care diagnostic methods as well as clinical recognition, they were often underreported. Studies investigating febrile illness during pregnancy differ on the main causal agent. In the Thai–Burmese population, the most common agent was malaria, compared to Laos, where dengue was more frequent [16,21]. However, nearly 5–10% of the patients had a co-infection with a rickettsial/rickettsial-like organism and up to 12% of patients had a rickettsial/rickettsial-like monoinfection [16,21,22]. In the past several years, there has been an increase in reports describing scrub typhus (an arthropod-borne disease caused by *Orientia tsutsugamushi*) during pregnancy associated with poor fetal outcomes [16,21,23]. Aside from a few observational studies, there are limited data regarding murine typhus during pregnancy. Unlike other tropical infections, such as leptospirosis and malaria, there are no data suggesting an increased incidence of MT in pregnancy [15,16]. 

In pregnancy, MT can mimic serious conditions that could impair the pregnancy if not addressed early. The differential diagnosis includes typical or atypical preeclampsia, given the presence of headache, thrombocytopenia, and abnormal liver function tests [6,21]. In up to 15% of patients, hemolysis, elevated liver enzymes, and low platelets syndrome (HELLP) can present without concomitant hypertension or proteinuria [14]. Chorioamnionitis and other infectious etiologies, such as those belonging to Toxoplasmosis, Others, Rubella, Cytomegalovirus, and Herpes viruses (TORCH) syndrome and arthropod-borne illnesses (e.g., scrub typhus, malaria, dengue, and Zika), could present similarly to MT. The disease should be high on the differential diagnosis when there is a history of exposure and knowledge of local epidemiology. Based on the data herein presented, MT does not seem to occur preferentially at any particular gestational age. Based on our findings, most patients present with a history of fever (>7 days) and remain hemodynamically stable. They present with headache, myalgias, arthralgia, which may be accompanied by a faint maculopapular rash. The most common abnormal labs are elevated liver enzymes and thrombocytopenia. The confirmatory diagnosis was mainly based on serology and PCR amplification of rickettsial DNA. Recently, the use of microbial cell-free DNA in human plasma has become an attractive option for the diagnosis of MT, given the potential of higher sensitivity than conventional molecular diagnostic tests such as PCR [17,24,25]. However, this novel modality is not widely available as a standard diagnostic test. 

In regard to the pregnancy outcome after these infections, natural history appears to be benign. In one study on the Thai–Burmese border, 33% of patients (n: 4) had poor prenatal outcome [15]. It is known that in resource-limited tropical countries, arthropod-borne infections in pregnancy are associated with poor outcomes [15,22]. In regard to MT during pregnancy, the relationship is less clear. Some observational studies in Southeast Asia have associated MT with low birth weight and stillbirths; however, the small sample makes it difficult to prove an independent association [21]. The pathogenesis of possible worse outcomes in pregnancy during rickettsial infections, if any, is unknown. Studies have speculated that endothelial damage causes a circulatory impediment, likely due to thrombotic occlusion or coagulopathy [26]. On the other hand, animal studies have shown no passage of the bacteria via placenta or through breastfeeding even when high blood concentrations of bacteria were noticed [6,27]. Based on our analysis, poor outcomes with *R. typhi* infections were noted only in limited-resource countries, where other cofounders such as poor nutrition, lack of perinatal care, and co-infections are present. In our review, there did not appear to be any differences in pregnancy outcomes and stage of pregnancy when patients were infected.

In the general population, first-line therapy for all rickettsioses, including MT, is tetracyclines. The use of tetracyclines in pregnancy has been an area of debate. Prior studies associating discoloration of developing permanent teeth in children and hepatic failure in pregnant women were based on the use of older formulations (e.g., tetracycline hydrochloride, oxytetracycline) but not doxycycline. Older tetracyclines caused discoloration of the primary teeth when taken during the 4th–5th month of gestation [6]. Large-scale observational studies and systematic review of the literature shows no increase in the incidence of congenital malformations with doxycycline [28,29]. Furthermore, case-control studies have shown that there was no significant difference in congenital abnormalities between the 2nd and 3rd month of gestation (the organogenesis period) with the exception of two cases with neural tube defect [30]. Despite all these recent data, the federal drug administration considers doxycycline a category D drug. Based on a systematic review of published data, the authors concluded that therapeutic doses of doxycycline are unlikely to have teratogenic risk, but more data are needed to strengthen the evidence for firmer recommendations [31]. 

Although previously reported success with macrolides has made them an attractive alternative during pregnancy [15], a recent prospective randomized trial based on 216 non-pregnant patients showed that doxycycline was superior to azithromycin for uncomplicated MT. Azithromycin was inferior based on fever clearance time, the time-temperature area under the curve, and frequency of treatment failure [32]. 

Reports documenting the treatment of MT during pregnancy overwhelmingly use agents other than doxycycline. One study, analyzing data of patients with MT and scrub typhus, showed that azithromycin was the most frequently administered therapy (66% of cases), while ciprofloxacin and others with no anti-rickettsial activity (i.e., ceftriaxone, ertapenem, and piperacillin–tazobactam) were used to treat the remaining cases. Although unable to adjust data based on the severity of the disease, treatment delay, pregnancy stage, or recurrent illness, patients with shorter fever clearance time were higher in the azithromycin group as compared to other non-tetracycline antibiotics [15]. There was a trend toward less poor neonatal outcomes in those treated with azithromycin as compared to those not treated at all, although the difference was not statistically significant. There was also a higher proportion of normal neonatal outcomes in those treated as compared to those who were not. Chloramphenicol has also been used in rickettsial diseases, but it should be noted that the drug crosses the placenta and is excreted in milk. Side effects that preclude its use are aplastic anemia and “gray baby syndrome” in neonates. 

Based on the data available, it is not possible to make a conclusion on the optimal treatment for MT in pregnancy. The data favor the safety of doxycycline during pregnancy, which is the first-line therapy for the disease in non-pregnant patients. There is no specific protocol for the prevention of murine typhus. Infection is thought to occur due to the inoculation of infected flea feces into wounds or mucous membranes. Therefore, avoidance of the vectors and their hosts is recommended [33]. Experts recommend controlling flea-infested pets and rat populations around residencies and businesses. There are numerous commercial products available for use on pets and around homes [34].

Passive immunization has been observed in the offspring of rats via colostrum or milk, regardless of the maternal antibody titer. However, the titers were usually undetectable by week 4 after birth [27]. Neonatal studies assessing the duration and protection of MT by maternal antibodies are lacking. There are currently no vaccines available to prevent MT, and prior efforts attempting to target other rickettsioses (e.g., Rocky Mountain spotted fever and louse-borne epidemic typhus) have been partially successful in animal models but inadequate for human use [1]. Vaccine development is imperative, as rickettsioses as a group have biological weapon potential given the aerosolized particle stability, high virulence, non-specific clinical diagnosis, inefficient diagnostic techniques, and low-level immunity [1]. 

The main limitation of this review is that the analyzed manuscripts were case reports and small prospective cohort studies; therefore, they are subject to recall and sampling bias as well as multiple confounding factors. Regarding our case reports, case #1 was defined as probable MT, not definite MT, as a fourfold change could not be observed. We were unable to test the specimens for PCR, as samples were not banked for a repository. Lastly, *R. felis* and *R. typhi* are sympatric and the possibility of some cases caused by *R. felis* instead of *R. typhi* is reasonable. However, prior studies in the Galveston area have shown a relatively low prevalence of *R. felis* in surveillance studies [4,7]. Furthermore, data from at least one person from Galveston during the disease’s local reemergence confirm that *R typhi* is circulating in humans [35].

Large-scale cohort studies exclusively studying MT during pregnancy are needed to fill gaps in knowledge regarding presentation, overall pregnancy outcome, and effectiveness of treatment during a particularly vulnerable state. This article provides an overview of MT presentation, diagnosis, and treatment in the pregnant population for the general clinician to consider, as it can mimic other pregnancy-related diseases requiring vastly different treatments.

## 6. Conclusions

Murine typhus in pregnancy is underreported. It can mimic other pregnancy-related pathologies. As noted in this literature review, confirmatory diagnosis is most often based on serology. The treatment varied, although doxycycline and azithromycin were the most frequently used. Overall, the pregnancy outcome seems to be favorable. More exclusive and large-scale studies are needed to learn more of murine typhus during pregnancy.

## Figures and Tables

**Table 1:** Published case series about murine typhus in pregnancy (1992–2020).

STUDY INFORMATION								
Author-Year	Graves-1992	Koliou-2007	Gutierrez-2010	Jolley-2010	McGready-2010	Chansamouth-2016	Tanabe-2019	Stafford-2020
Location (Country)	Australia	Cyprus	USA	USA	Thai-Burmanese border	Laos	USA	USA
Number of patients	1	1	1	1	14	15	2	2
**MATERNAL HEALTH**								
Age (mean/range)	17	30	26	33	27 (16–36)		28 (20–36)	29 (24–34)
Gestational Age (mean/range)	23	30	26	32	21 (7–39)	39 (35–40)	21 (12–30)	22 (13–31)
Maternal comorbidities (n) **			✕	✕	✓ (5)	✓ (5)	✓ (1)	✕
Prior pregnancies (n)	✕		✕	✕	✓ (11)		✓ (1)	✓ (1)
Risk factor for MT				cats, opposums			cats	pets and fleas
**CLINICAL COURSE**								
Headache (n)	✓	✓	✓	✓	✓ (13)		✓ (2)	✓ (2)
Rash (n)	✓	✓	✕	✓	✕		✓ (1)	✓ (1)
Fever (n)	✓	✓	✓	✓	✓		✓ (2)	✓ (2)
Transaminitis (n)	✓	✓	✓	✓			✓ (2)	✓ (2)
Hyponatremia (n) #	✕	✕	✓				✓ (2)	✕
Thrombocytopenia (n)	✓	✕	✕	✓			✓ (2)	✓ (1)
Diagnosis	serology	serology	serology	serology	serology, PCR	serology, PCR	serology	microbial cell-free DNA
Treatment	erythromycin	erythromycin	ampicillin	azithromycin	azithromycin	azithromycin	azithromycin, doxycycline	azithromycin, doxycycline
**FETUS HEALTH**								
Born at term (n) ***	✓ (1)	✓ (1)	✓ (1)	✓ (1)	✓ (10)	✓ (8)	✓ (2)	✓ (1)
Poor outcome (n) *	✕	✕	✕	✕	✓ (4)	✓ (2)	✕	✕
**REFERENCE**	11	12	13	14	15	16	n/a	17

**Footnote**: ✓: present ✕: absent.* Poor outcome: stillborn, abortion, prematurity (<35 weeks), and fetal growth restriction (birth weight < 10th percentile). ** Comorbidities: diabetes mellitus, hypertension, substance abuse, tobacco use, co-infections, dyslipidemia, heart disease, eclampsia/preeclampsia. *** TERM: 37–42 weeks. # Hyponatremia < 134 meq/dL, Absolute thrombocytopenia PLT < 150,000 cells/cm^3^, Transaminitis ALT > ULN (40). (BLANK): Information not given. The table was filled based on the best information provided by the article. Totals do not equal 100% as some patients’ data were missing.

## Data Availability

The data presented in this study is contained within the article.
